# Immunotherapy for High-Grade Gliomas

**DOI:** 10.3390/cancers17111849

**Published:** 2025-05-31

**Authors:** Nishika Karbhari, Kelsey M. Frechette, Terry C. Burns, Ian F. Parney, Jian L. Campian, William G. Breen, Ugur T. Sener, Eric J. Lehrer

**Affiliations:** 1 Department of Neurology, Mayo Clinic, Rochester, MN 55905, USA; karbhari.nishika@mayo.edu (N.K.); sener.ugur@mayo.edu (U.T.S.); 2 Department of Medical Oncology, Mayo Clinic, Rochester, MN 55905, USA; campian.jian@mayo.edu; 3 Department of Radiation Oncology, Mayo Clinic, Rochester, MN 55905, USA; frechette.kelsey@mayo.edu (K.M.F.); breen.william@mayo.edu (W.G.B.); 4 Department of Neurosurgery, Mayo Clinic, Rochester, MN 55905, USA; burns.terry@mayo.edu (T.C.B.); parney.ian@mayo.edu (I.F.P.)

**Keywords:** glioma, glioblastoma, high-grade glioma, immunotherapy, immunology

## Abstract

**Background:** High-grade gliomas (HGGs), particularly glioblastoma (GBM), are associated with exceptionally high mortality and inevitable recurrence. In considering novel treatment options for these devastating diseases, immunotherapies represent promising candidates. Immunotherapies have demonstrated efficacy for several advanced tumors outside the central nervous system, highlighting a potential role for these agents in treating HGGs. However, multiple challenges to immunotherapy efficacy have tempered therapeutic benefit in practice, including local and systemic immunosuppression, intratumoral heterogeneity, and various mechanisms of intrinsic and acquired resistance. In the past 30 years, diverse immunotherapeutic subclasses have been assessed for benefit against HGGs. **Methods:** We performed a PubMed search for randomized clinical trials performed within the last 30 years evaluating the following immunotherapy agents for high-grade gliomas: immune checkpoint inhibitors, vaccines, oncologic viruses, cytokines, and CAR T-cells. The present review offers a critical analysis of key pre-clinical and clinical trials that have shaped the immunotherapy landscape for high-grade gliomas over the past two decades. **Results/Conclusions:** Across the different immunotherapeutic methods and modalities explored thus far, a recurring theme emerges: while therapeutic strategies with a compelling conceptual basis are continually under development and even demonstrate a benefit in preclinical and early-phase trials, larger and later-phase trials consistently fail to produce concordantly significant outcomes. To date, no large-scale clinical trial has demonstrated a benefit of sufficient consequence to change practice. Continued critical appraisal of the strengths and pitfalls of prior investigative work, optimization of treatment development and delivery, and innovative approaches to combination therapy design will collectively be integral to future therapeutic advancement.

## Simple Summary

High-grade gliomas (HGGs), particularly glioblastoma, remain among the most lethal cancers, with limited treatment success and high recurrence rates. Immunotherapy has shown promise in other cancers and is being explored and refined as a treatment option for HGGs. This review examines randomized clinical trials from the past 30 years involving various immunotherapy strategies—including checkpoint inhibitors, vaccines, oncolytic viruses, cytokines, and CAR T-cells. While early-phase studies often show encouraging results, larger trials have consistently failed to deliver significant clinical benefits. The findings highlight the need for improved preclinical models, better trial design, and innovative combination therapies to enhance future outcomes in HGG immunotherapy.

## 1. Introduction

Gliomas are tumors derived from glial cells of the brain, which provide nutritional, metabolic, and structural support to neurons. From 2016 to 2020, gliomas accounted for 26.3% of all primary central nervous system (CNS) tumors [1]. Of the gliomas, glioblastoma (GBM) is the most aggressive entity, with a WHO grade 4 designation and a particularly poor prognosis. GBM is also the most common primary malignant brain tumor, comprising 14.2% of all primary CNS tumors, and 50.9% of all primary malignant CNS tumors [1]. The treatment standard for GBM was established in a landmark 2005 study by Stupp and colleagues, showing a survival benefit associated with the addition of temozolomide to maximal safe resection and radiation therapy [2]. Methylation of the MGMT (O6-methylguanine-DNA methyltransferase) promoter, which inhibits DNA damage repair mechanisms, is associated with increased sensitivity to temozolomide. To date, one additional intervention has shown a clinically significant survival benefit in GBM is the Tumor Treating Fields (TTF) device, associated with increased overall survival by 4–5 months when added to standard of care therapy [3]. Despite these interventions, outcomes for GBM remain dismal, with a 5-year survival rate of 6.9% [1]. Without exception, the natural history of GBM entails recurrence. Novel treatments with the potential to meaningfully extend survival are thus of critical and compelling need.

Per the 2021 WHO criteria, a wild-type isocitrate dehydrogenase (IDH) gene is now required for the diagnosis of GBM. Recognizing the importance of such molecular features, the diagnostic criteria for GBM has recently expanded to encompass certain genetic alterations that are known to confer a worse prognosis, thus elevating the diagnosis of a tumor that may otherwise appear lower grade by histologic criteria to a true Grade 4 GBM [4]. These alterations include TERT promoter mutations, gain of chromosome 7 and loss of chromosome 10, and EGFR amplification [4].

In considering other treatments with the potential to improve clinical outcomes, immunotherapies have been identified as strong potential candidates. Immunotherapies are a class of treatments with the unifying theme of enhancing the ability of the host immune system to reach, recognize, and kill tumor cells. This group of treatments encompasses checkpoint inhibitors, oncolytic viruses, vaccines, and chimeric antigen receptor therapies (CAR-T). Each subclass will be discussed in detail in this review.

Finally, in developing strategies to maximize the therapeutic potential of immunotherapies, obstacles to achieving and determining efficacy must be considered. Regional molecular diversity across a tumor, intrinsic and acquired resistance to treatment, tumor-associated immunosuppression, and longitudinal biologic assessments of therapeutic response are all relevant factors for consideration.

## 2. Challenges and Considerations for Immunotherapy Efficacy

The rationale for harnessing immunotherapy for the treatment of high-grade gliomas (HGGs) is supported by an array of success across various non-CNS solid malignancies. Checkpoint inhibitors have exhibited exceptional success in the treatment of patients with advanced melanoma [5,6] and efficacy in other advanced solid cancers [7], and CAR-T cell therapy has shown promise in hematologic malignancies [8]. Despite the many advancements in developing immunotherapies for HGGs, significant challenges to efficacy and durability of these treatments still exist. In the present section, we will describe these challenges and note important considerations for continued therapeutic development.

### 2.1. Immunosuppressive Tumor Microenvironment

One of the most significant challenges to immunotherapy efficacy is the immunosuppressive tumor microenvironment (TME). The brain is characterized by a significantly reduced population of T cells compared to other organs, and the GBM TME is composed mainly of macrophages which often serve a tumorigenic function in the vicinity of tumor cells [9,10,11]. Among these tumor-associated macrophages (TAMs), bone marrow-derived macrophages (BMDMs), estimated to constitute approximately 70% of the GBM TAM population, are enriched near vascular structures and necrotic regions of tumor [12]. In contrast to TAMs of microglial origin, BMDMs upregulate immunosuppressive cytokines, thus assuming an M2-like phenotype and facilitating host immune evasion [12]. These immunosuppressive cytokines in turn stimulate the oncogenic STAT3 pathway, which hinders the anti-tumor immune response and promotes tumor progression [13]. In fact, a preclinical model combining radiation therapy (known to induce STAT3 activation) with a STAT3 inhibitor was found to be associated with enhanced dendritic and T-cell interactions, and improved survival time [14].

Various tumor cell metabolites also facilitate immunosuppression in the TME [11]; perhaps most well-recognized example in the setting of gliomas is the D enantiomer of 2-hydroxyglutarate [15]. These findings suggest a role for therapies targeting cellular and biochemical properties with specific inhibition of the BMDM population of TAMs and immunosuppressive metabolic pathways, respectively, as avenues to enhance anti-tumor immunogenicity. Indeed, several preclinical studies have explored these approaches with promising results [16,17,18].

Glioma stem cells (GSCs) represent both a potential challenge and opportunity in the context of immunotherapy. Due to their ability to remain in a quiescent state, GSCs have been increasingly recognized as a cell population capable of mediating resistance to standard chemotherapeutic agents and radiation [19,20]. GSCs also play a role in local immunosuppression within the TME [21]. Targeting GSCs via an immunotherapeutic approach dependent on antigenic features rather than a proliferative phenotype may therefore offer a strategy for killing tumor cells that survive standard of care chemoradiation. Preclinical work has suggested that dendritic cell vaccines derived from human GSCs can induce cytotoxic T lymphocyte activity [22]. Additional work studying this important cell population will likely enable the development of more efficient and effective immunotherapeutic agents and combination regimens in the future.

### 2.2. Immunosuppressive Molecules and Markers

Notable components of the GBM TME that facilitate immunosuppression include TGF-β2, PGE-2, IL-6, and IL-10. TGF-β2 is strongly upregulated in GBM cells [23,24]. By suppressing HLA-DR antigen expression on human malignant glioma cells [25] and the expression of key cytolytic gene products through which cytotoxic T cells exert tumor cytotoxicity [26], TGF-β2 promotes immune evasion and suppression. Signaling through the TGF-β pathway further suppresses cytotoxic immune activity by enhancing regulatory T cell-mediated inhibition of CD8 T-cell populations [27]. PGE-2 is abundant in the GBM TME [28] and exerts a range of immunosuppressive effects. Elevated levels of PGE-2 inhibit the activity of lymphokine-activated killer cells [29], downregulate expression of MHC-II molecules on antigen-presenting cells [30], and hinder T-cell activation [31,32]. IL-6 induces myeloid PD-L1 expression in GBM, and disruption of IL-6 signaling in orthotopic murine glioma models decreased local and peripheral myeloid-mediated immunosuppression and promoted antitumor immunoactivity [33]. IL-10 is overexpressed in high-grade gliomas [34,35], and tumor-associated macrophages (TAMs) are an important source of IL-10 [35,36]. IL-10 has been shown to induce TAM transition to an anti-inflammatory phenotype in vitro, both independently [37] and in synergy with IL-4 [38].

The checkpoint protein PD-L1 is upregulated in GBM [39], a feature associated with negative prognosis due to the ensuing immune evasion [40,41]. PD-L1 expression is positively correlated with immunosuppressive cell levels in the TME and negatively correlated with TME cytotoxic immune cell levels [41]. High PD-L1 expression is also significantly correlated with TAM transition to the anti-inflammatory M2 phase [41]. Peripherally, PD-L1 expression is associated with an increased fraction of regulatory T cells in GBM [42]. These patterns of PD-L1 expression and immune regulation support the continued development of checkpoint inhibitors to treat GBM.

Indoleamine 2,3-dioxygense 1 (IDO-1) is an inducible tryptophan catabolic enzyme present on various cancerous cells including GBM. IDO-1 plays an important role in immune regulation, with implications for overall clinical outcomes. Preclinical GBM models have demonstrated the ability of IDO-1 to mediate immunosuppression through inhibition of CD8+ T cells [43]. Additionally, elevated GBM IDO-1 expression has been associated with increased levels of immunosuppressive regulatory T cells and myeloid-derived suppressor cells [44]. Higher levels of IDO-1 transcription have also been shown to correlate with poor patient prognosis in GBM [45].

The CD47-signal regulatory protein alpha (SIRPα) regulates the function of glioma-associated microglia and macrophages (GAM) [46], the predominant cell population in the tumor microenvironment. CD47 is frequently overexpressed by GBM and acts as a phagocytosis immune checkpoint, facilitating tumor cell evasion of host phagocytes and GAMs. In murine models, CD47 blockade restored GAM antitumoral phagocytic function [47]. In a study aiming to exert immunoactivity while overcoming inherent immunosuppression, a CAR T cell product against EGFRvIII was engineered to constitutively secrete a signal regulatory protein gamma (SIRPγ)-related protein (SGRP) with a high affinity for CD47 [48]. The high binding affinity of the SGRP used in this study for CD47 was also important in overcoming antigen escape, an otherwise evasive barrier to immunotherapy efficacy that is discussed in detail below. In an orthotopic EGFRvIII-mosaic GBM mouse model, anti-EGFRvIII-SGRP CAR T cells eliminated tumor cells in vivo, demonstrating superiority to a CAR T product that targeted EGFRvIII alone and resulted in tumor cell persistence. In a subcutaneous mouse model of CD19+ lymphoma, anti-CD19-SGRP CAR T was likewise found to be superior to conventional anti-CD19 CAR T therapy [48]. While these preclinical data are encouraging, results from an upcoming clinical trial will be necessary for safety confirmation and efficacy assessment of this novel product.

### 2.3. Systemic Immunosuppression

Systemic immunosuppression can be profound in patients with GBM, presenting an important barrier to immunotherapy success. Among the many manifestations of systemic immunosuppression in this population is a global reduction in T-cell counts and function [49]. CD4 T-cell counts in GBM patients can reach nadirs comparable to those of patients with acquired immunodeficiency syndrome [50]. Several mechanisms underlie this, including large-scale bone marrow T cell sequestration through a tumor adaptive strategy employing disrupted S1P1 signaling, resulting in dysregulated lymphoid migration [51]. Regulatory T cells often constitute an increased fraction within the remaining CD4 T-cell compartment, further augmenting immunosuppression [50]. Additionally, myeloid-derived suppressor cells (MDSCs) are significantly elevated in the peripheral blood of patients with GBM, and increased MDSC levels are associated with poor prognosis in patients with recurrent GBM [52]. Downstream immunosuppressive effects from MDSC tumor infiltration include T cell suppression [53,54] and reductions in tumor-infiltrating lymphocytes [55]. Intratumoral density of TAMs and MDSCs has also been shown to inversely correlate with patient’s survival [56].

### 2.4. Antigen Escape

Antigen escape occurs when an immunotherapy product is unable to recognize a tumor antigen. This may be due to a variety of mechanisms, among which are subclonal expansion of a cell population with low antigen expression as may occur after CAR T cell treatment, tumor cell lineage switch, and CAR-T capture of tumor antigens through a process termed trogocytosis, which in turn hinders CAR-T antigen recognition [57]. While antigen escape is often observed in the context of T cell therapy, it presents a notable potential barrier to the success of vaccine therapies as well. Antigen escape was highlighted in the study of Ridopepimut where loss of target EGFRvIII expression was ultimately observed independently of vaccine treatment, contributing to therapy failure [58]. Other EGFRvIII-targeting products for GBM have also been limited by antigen escape [59,60]. Checkpoint inhibitor treatment for various tumors can also precipitate antigen escape by inducing down-regulation of the targeted antigen [61].

Combination treatments and multimodal therapy regimens represent important strategies for overcoming antigen escape, by targeting more than solely a single, potentially dynamic antigen. A stronger understanding of the mechanisms underlying antigen escape and contributing to recent treatment failure has driven the development of novel therapies designed to overcome this important obstacle. These include CAR T products with activity against more than one target [48,62,63] and combination immunotherapy or multimodal regimens, as discussed in detail above. More comprehensive characterization of the molecular basis of antigen escape will inform the development of novel therapeutic agents and combinations with efficacy in overcoming this critical phenomenon.

### 2.5. Intratumoral Heterogeneity

Drug efficacy within various parts of a tumor may differ due to intratumoral heterogeneity, characterized by molecular diversity and subclonal variation across different tumor regions. Several groups have used single-cell and bulk RNA sequencing to transcriptionally profile GBM, demonstrating variability in oncogenic molecular pathways, immune response, stress response, and hypoxia within individual tumor samples [64,65,66]. Variability in epigenetic features including MGMT promoter methylation also contributes to overall heterogeneity [67]. Intratumoral heterogeneity poses a significant challenge to therapeutic efficacy, noting especially the development of distinct subclonal cell populations within a tumor with molecular and physiologic differences that confer differential sensitivity to treatment [68,69,70]. Additionally, following the administration of certain drugs (notably temozolomide), treatment-induced hypermutation can further amplify regional genetic differences within a tumor [11,71].

Acknowledging the extent of intratumoral heterogeneity in GBM has led to an increased emphasis on combination therapies, with the intent of targeting multiple potential tumorigenic features in tandem [69,72]. Other strategies developed in an attempt to overcome heterogeneity include autologous vaccines, as described in detail above [73], which are designed to account for the spectrum of variation across a particular tumor. Going forward, the continued development of therapeutic approaches addressing the substantial and nuanced variability within high-grade gliomas will be critical.

### 2.6. Adaptive and Acquired Resistance

In addition to the above factors that confer intrinsic resistance to treatment, adaptive and acquired resistance to treatment are important obstacles to immunotherapy efficacy. Adaptive resistance arises from a reduction in potential therapeutic targets, while acquired resistance results from the accumulation of genetic alterations under immunologic pressure [69,74]. An important example of immunotherapy resistance was highlighted in a study that longitudinally studied 66 GBM patients during and after treatment with a single-agent PD-1 inhibitor. Genomic and transcriptomic analyses showed that PTEN mutations were significantly enriched in non-responders, and RNA sequencing suggested these mutations may contribute to an immunosuppressive microenvironment [75]. Immunotherapy is also associated with T cell exhaustion, an important functional consequence of long-term immunotherapy treatment that ultimately mediates treatment resistance. T cell exhaustion, originally reported in chronic viral infections, is a state in which the T cell responsiveness is decreased under conditions of chronic antigenic exposure [76]. T cell exhaustion is particularly severe in GBM, and is associated with immune checkpoint upregulation and a distinct transcriptional program [77]. These resistance mechanisms again underscore the potential benefit of combination therapy regimens, where immunologic selective pressure from any individual agent is relatively reduced. Longitudinal response assessment, as described in detail below, may also help characterize mechanisms of resistance more comprehensively and in real-time.

### 2.7. Longitudinal Response Assessment

In evaluating treatment response, outcomes that accurately reflect therapeutic efficacy must be defined. Most studies included in the present review and within the literature as a whole identify progression-free survival (PFS) as an outcome measure. However, the challenge of radiographically differentiating true progression from pseudoprogression can confound assessment of PFS. Radiographic assessment alone is also insufficient for characterizing treatment effects on underlying tumor biology. In order to gauge the true efficacy of immunotherapeutic agents, accurate and precise measures of response are imperative. Assessment across various timepoints may further enhance the sensitivity of analysis. Accordingly, many investigators have advocated for serial tissue sampling [78,79]. Serial tissue sampling can enable dynamic analysis of therapeutic targets, tumor microenvironmental composition, and mechanisms underlying treatment resistance [79]. Real-time feedback provided by these assessments can help characterize the biological underpinnings of clinical and/or radiographic response or progression at any given timepoint and may even enable earlier consideration for treatment changes. Recognizing the value of serial tissue sampling, various groups have focused on increasing implementation of this method. The international Glioma Longitudinal Analysis (GLASS) Consortium was established to increase acquisition of molecular tumor information, with important implications for the selection of targeted therapy [80]. The multi-institutional consortium Breakthrough Cancer launched an early-phase oncolytic virus trial emphasizing serial biopsies for longitudinal assessment (NCT03152318), discussed in detail below (see Section 5.2.).

Similarly, serial cerebrospinal fluid (CSF) assessment, or liquid biopsy, can serve an important role in determining treatment response. The generally protein-deficient nature of CSF compared to plasma yields an improved signal-to-noise ratio for biomarker measurement [81]. However, technical approaches to CSF sampling can be nuanced. Intrinsic proteomic differences between various CSF compartments such as brain and spine have underscored the need for accurate sampling, which is enhanced within tumor-contacting fluids [81]. Devices such as the Ommaya reservoir may facilitate longitudinal intracranial CSF sampling for metrics of treatment response and progressive disease, though results may be confounded by sample yield variability and local responses to surgery [82]. While liquid biopsy presents a potentially promising avenue for therapeutic response monitoring, advancements in serial sampling techniques will be important to the continued development of this tool for longitudinal response assessment.

### 2.8. Blood–Brain Barrier Penetration

Historically, a key obstacle to drug delivery in central nervous system pathologies has been the blood–brain barrier (BBB). In the setting of immunotherapy, drug penetration through the BBB is in fact less of a consideration. The goal of immunotherapy is to induce a robust peripheral immune response, generating immune modulators that are naturally capable of traversing the BBB. This justifies the intravenous or intradermal delivery routes that are most commonly employed for immunotherapy administration, even when the target site is the central nervous system. Still, certain strategies have been devised to more precisely target the tumor site, including direct therapeutic administration into the tumor, as performed with several of the therapies discussed in the present review [62,83,84,85,86,87,88], convection-enhanced delivery [84,89], focused ultrasound [90], and delivery via nanoparticles [91,92,93,94].

## 3. Methods for Clinical Trial Selection

To select trials for description and analysis in this systematic review, a series of PubMed searches were conducted. Publication dates over a 30-year period (15 February 1995 to 15 February 2025) were selected for all searches. Article types ‘Clinical Trial’ and ‘Randomized Controlled Trial’ were selected for all searches. Ten separate searches were performed, with two searches per immunotherapy class, queried as follows: (1) ‘Immune Checkpoint Inhibitor for Glioblastoma’; ‘Immune Checkpoint Inhibitor for High-Grade Glioma’; (2) ‘Vaccine for Glioblastoma’; ‘Vaccine for High-Grade Glioma’; (3) ‘Virus for Glioblastoma’; ‘Virus for High-Grade Glioma’; (4) ‘Cytokine for Glioblastoma’; ‘Cytokine for High-Grade Glioma’; (5) ‘CAR for Glioblastoma’; ‘CAR for High-Grade Glioma’. This search collectively returned 788 results. Results were manually filtered to exclude studies that were not clinical trials, redundant, or not pertinent to high-grade gliomas. The authors additionally included a small set of studies relevant to the present topic that were not returned by the above search.

## 4. Checkpoint Inhibitors

The purpose of immune checkpoint inhibitors is to activate the host immune system to recognize cancer cells. Immune checkpoint molecules are present on the surface of certain host immune cells and mediate the natural function of immunotolerance to the body’s own tissues. When immune checkpoints recognize a host tissue, an antigen-binding reaction occurs that deactivates T cells, protecting the tissue from an unwarranted immune response. In the context of cancer, however, this phenomenon is exploited by malignant cells. Antigens on the surfaces of these cells bind the checkpoint molecules, triggering a downstream reaction that results in T cell neutralization, and in turn facilitating host immune invasion. Checkpoint inhibitors are designed to serve as a decoy, binding the tumor’s checkpoint antigens with high affinity and specificity [95], thus essentially outcompeting T cells and preserving their function. Alternatively, checkpoint inhibitors may bind T cells themselves, protecting the binding site from interacting with the tumor. Important investigative work evaluating checkpoint inhibitors will be described in this section. Active and recently completed clinical trials of immune checkpoint inhibitors are summarized in Table 1.

The PD-1/PD-L1 (programmed cell death protein-1/programmed death ligand-1) is a notable checkpoint interaction against which various inhibitors have been developed and trialed. PD-1 is expressed on host immune cells, and PD-L1 on tumor cells. Pembrolizumab is a monoclonal antibody directed against PD-1 that has gained prominence as a treatment for other systemic cancers, notably melanoma, non-small cell lung cancer, and certain gastrointestinal cancers [7,96,97]. Pembrolizumab was also studied in GBM. In the open-label, non-randomized KEYNOTE basket trial for solid tumors harboring PD-L1, the recurrent GBM cohort enrolled 26 adult patients who received pembrolizumab every 2 weeks for up to 2 years. Patients in this cohort demonstrated tolerable safety, median progression-free survival (mPFS) of 2.8 months, median overall survival (mOS) 13.1 months, and 12-month median survival of 58% [98]. Another important PD-1 inhibitor is the monoclonal antibody nivolumab. Perhaps the most notable studies evaluating nivolumab in recurrent GBM are the CheckMate trials. CheckMate 143 was the first Phase 3 clinical trial evaluating nivolumab in recurrent GBM and compared the efficacy of nivolumab (treatment arm) to bevacizumab (control) across a total of 369 patients at first recurrence. At a median follow up of 9.5 months, mOS was 9.8 months for nivolumab versus 10 months for bevacizumab. Though the objective response rate was reported as higher in patients treated with bevacizumab (23.1%) compared to nivolumab (7.8%), it is unclear whether some degree of pseudo-progression may have confounded results in the nivolumab group [99]. Additionally, the evidence supporting selection of nivolumab as the agent for Phase 3 level investigation was questionable, noting that most earlier phase studies had assessed outcomes related to pembrolizumab.

PD-1 inhibitor-based combination regimens have been studied as well. A Phase 1 study of pembrolizumab both as monotherapy and in combination with bevacizumab for the treatment of recurrent GBM showed no benefit to immune checkpoint inhibitors in both the monotherapy and combination therapy groups (mOS 10.3 months for pembrolizumab monotherapy vs. 8.8 months for combination treatment) [100]. Two other Phase 3 iterations of CheckMate investigated nivolumab in newly diagnosed GBM. In CheckMate 548, 716 patients with MGMT promoter methylation received standard of care treatment of temozolomide-based chemoradiotherapy, combined with either nivolumab or placebo control. Nivolumab did not improve survival (mOS 28.9 months nivolumab vs. 32.1 months placebo; PFS 10.6 months for nivolumab vs. 10.3 months for placebo) [101]. CheckMate 498 studied 560 newly diagnosed patients with MGMT promoter unmethylated GBM. Patients received radiation therapy in combination with either nivolumab or temozolomide. Nivolumab was not associated with improved survival (mOS 13.4 months for nivolumab vs. 14.9 months for temozolomide) [102].

Various strategies have been employed in an effort to elucidate a benefit from anti-PD-1 therapies. In addition to different combination regimens, variable timelines of administration have been studied. In a trial evaluating neoadjuvant plus adjuvant treatment of pembrolizumab compared to adjuvant treatment alone for 35 patients with recurrent GBM, mOS for neoadjuvant plus adjuvant pembrolizumab was 13.7 vs. 7.5 months for adjuvant pembrolizumab alone, with mPFS 3.3 vs. 2.4 months, respectively [103]. However, subsequent efforts to reproduce these results were not successful. In a stage 2, single-arm expansion cohort evaluating neoadjuvant pembrolizumab for surgically accessible recurrent GBM, 6-month PFS was 19.5%, and OS and PFS were similar to historical controls [104]. A similar study was conceptualized to evaluate neoadjuvant nivolumab in recurrent GBM. In this single-arm study of 30 patients with recurrent GBM who received a presurgical dose of nivolumab followed by postsurgical treatment, mOS was 7.3 months. [105] While mixed results were observed across these studies, the small sample sizes included in both necessitate cautious interpretation and contextualization. As has previously been noted, many patients with recurrent GBM are not amenable to surgery upon recurrence; factors generally associated with surgical eligibility include smaller tumor size, younger patient age, higher performance status, and more extensive initial resection. [106,107]

CTLA-4 (cytotoxic T-lymphocyte associated protein-4) is another checkpoint molecule that has served as an important therapeutic target. CTLA-4 is present on T cell surfaces and, when bound, leads to T cell suppression. Ipilimumab is a CTLA-4 inhibitor that has been studied in various trials for potential efficacy across a range of tumor types. Trials thus far have examined ipilimumab in combination with temozolomide for recently diagnosed GBM [108], or in combination with nivolumab for recurrent GBM [109], and have not shown a benefit associated with ipilimumab compared to temozolomide or nivolumab alone in these respective settings. Interestingly, a Phase 1 study of 27 patients with recurrent GBM did show a benefit associated with intracerebral administration of nivolumab and ipilimumab. In this study, patients were treated with neoadjuvant intravenous nivolumab, followed by maximal safe resection, followed by adjuvant intracerebral administration of either ipilimumab alone or ipilimumab plus nivolumab. Because of the difference in sample size between the ipilimumab monotherapy group (*n* = 3) and the combination therapy group (*n* = 24), a historical control group was designated, consisting of patients treated with axitinib, avelumab, or lomustine in prospective clinical trials. Patients on the study compared favorably to historical controls. No dose-limiting toxicities were observed, and the incidence of immune-related adverse events through this intracerebral administration was in fact found to be lower than that associated with intravenous regimens [110]. More investigation in later phase trials will be necessary to evaluate the true efficacy of this regimen in treating GBM.

Novel targets for checkpoint inhibitors include TIM-3, TIGIT, and LAG-3 [111]. TIM-3 and TIGIT are coinhibitory molecules expressed on immune cells. Anti-Lag-3 (relatlimab) plus nivolumab is being compared to lomustine (standard of care) in a randomized Phase 2 clinical trial for recurrent glioblastoma (NCT06325683). TIM-3 is currently under investigation in a Phase 1 trial in combination with stereotactic radiosurgery (SRS) and a PD-1 inhibitor (NCT03961971). TIGIT is being evaluated in an ongoing phase 0/1 trial for recurrent GBM (NCT04656535). Inhibition of various immunoregulatory metabolites has also been studied in combination with checkpoint inhibitors. CD73 is a molecule that facilitates the degradation of adenosine monophosphate (AMP) to adenosine. Oleclumab, or MEDI9447, is an antibody against CD73 that inhibited lymphocyte suppression by AMP in preclinical studies [112], and has since been evaluated in combination with the anti-PD1 agent durvalumab in a clinical trial for adults with select solid tumors (NCT02503774).

Newer strategies for evaluating checkpoint inhibitors have focused on combination regimens, with the intent of identifying benefit where monotherapy has previously failed. One such study of 21 patients with recurrent high-grade gliomas evaluated checkpoint inhibitor therapy combined with SRS and found no grade 3 toxicities in the entire cohort, suggesting feasibility and tolerability of combined modality treatment in a heavily pretreated population [113]. Several ongoing or recently completed clinical trials have been designed accordingly, aiming to harness pharmacologic or multimodal synergy. These trials are summarized in Table 1. Among these are a current Phase 2 trial studying pembrolizumab in combination with temozolomide and the Poly ADP-ribose polymerase (PARP) inhibitor olaparib for recurrent GBM (NCT05463848), a Phase 1b/2 trial testing pembrolizumab in combination with surgery and stereotactic radiation in recurrent GBM (NCT04977375), and a Phase 1/2 trial studying pembrolizumab combined with laser interstitial thermal therapy (LITT) for recurrent GBM (NCT03277638). Clinical trials evaluating combination treatment strategies with checkpoint inhibitors are likewise underway, including a Phase 2 trial studying the PD-1 inhibitor retifanlimab in combination with bevacizumab and hypofractionated radiation for recurrent GBM (NCT06160206), and a Phase 2 trial combining pembrolizumab with a long-acting recombinant interleukin-7 (NT-I7) for recurrent GBM (NCT05465954).

## 5. Oncolytic Viruses

### 5.1. Background

Upon recognition that oncolytic viruses (OVs) predominantly exert an immunostimulatory mechanism as opposed to the less extensive function of direct oncolysis, OVs have become a significant investigative focus in the field of immunotherapeutics. Different types of viral therapies have been developed to exploit this immunogenic effect, including genetically unmodified agents with natural tumor tropism, genetic modification to achieve tumor tropism, and genetic modification for other purposes relating to immunoregulation or overcoming treatment resistance.

OVs are derived from attenuated species grown in the lab [114]. They are developed to exhibit high specificity for cancer cells. This typically involves a 2-step genetic engineering procedure: (1) Retargeting, which involves the addition of a new, cancer-specific ligand to the virus; (2) Detargeting, in which nonspecific binding to non-cancerous cells is blocked [115]. Alternatively, a biochemical approach using antibody-virus interactions or covalently coupled cross-links, among other mechanisms, may be employed; however the pitfall to this approach is that the chemical modification is not transmitted to viral progeny, which default to their intrinsic genetic profile [115].

Once the virus enters the cancer cell, oncolysis occurs through either a direct or indirect approach. In direct oncolysis, viral replication within the cancer cell and the subsequent increase in viral load directly mediate cell lysis. In indirect oncolysis, high viral load following replication triggers the release of damage-associated molecular pattern (DAMPs) and pathogen-associated molecular pattern (PAMPs), in turn facilitating immune cell recruitment by major histocompatibility complex (MHC) and antigen-presenting cells (APCs), and ultimately promoting apoptosis [114]. Evidence suggests that viruses may exert an additional anti-tumor mechanism by disrupting tumor blood supply, which undermines vital nutritional support and oxygenation to the tumor, resulting in extensive cell death [116]. In GBM, neurotropic viral therapies additionally serve the distinctive role of stimulating the transition of a notoriously ‘cold’ tumor microenvironment to one that is ‘hot’, or hospitable to immune cells [117]. Several clinical trials have evaluated the efficacy of oncolytic virus therapies for GBM, either alone or in combination with immunotherapies or chemotherapy (Table 2). Some of the most prominent therapeutic viruses and trials for high-grade gliomas are highlighted below.

### 5.2. Herpes Simplex Virus

HSV has been considered a promising therapeutic agent for central nervous system tumors given the natural neural tropism of the virus [118]. Modifications to the virus have occurred over time with the goal of enhancing safety and efficacy. Of recent interest is M032, a selectively replicative, second-generation oncolytic herpes virus that induces IL-12 expression. M032 was studied in an open-label, dose-escalating Phase 1 clinical trial in patients with recurrent GBM, anaplastic astrocytoma, or gliosarcoma (NCT02062827). 21 patients received the treatment. Though two grade 3 and two grade 4 adverse events occurred in a patient with a large tumor, the adverse event profile was overall acceptable, with no dose-limiting toxicities observed at the maximum dose. While the trial is ongoing, preliminary data indicated a favorable response in some patients, with median post-treatment survival of 9.38 months [119]. An additional Phase 1/2 clinical trial is studying M032 in combination with pembrolizumab for patients with newly diagnosed or recurrent GBM, anaplastic astrocytoma, or gliosarcoma; this trial is currently recruiting (NCT05084430).

G47Δ, a triple mutated, third-generation oncolytic HSV-1, was developed to enhance the anticancer benefit of its precursor, the second generation HSV-1 product G207. The enhanced antitumor activity arises from an amplified host antitumor immune response, and increased viral load resulting in increased pathogenic effect [120]. G47Δ was initially tested in a Phase 1/2 study for recurrent GBM completed in 2014 in Japan [121], paving the way for a 2015 Phase 2 study involving stereotactic injection of G47Δ into the brain tumor of 19 adult patients. A maximum of six injections could be received. The primary endpoint was met (1-yr survival rate of 84.2%), leading to early trial termination. The mOS was 20.2 months following G47∆ administration and 28.8 (20.1–37.5) months from the initial surgery. The best overall response, measured at 24 months since the final G47∆ administration, was PR in one patient and stable disease in 18 patients [83]. These compelling results led G47∆ to be the first oncolytic virus product approved in Japan.

The oncolytic herpes virus CAN 3-110 was developed to retain an important neovirulence gene transcribed by a promoter for the gene *nestin* [122]. Though overexpressed in GBM, *nestin* is normal in healthy brain tissue, enabling selective tumor replication. In a first-in-human Phase 1 trial of 41 patients with recurrent HGG or GBM, CAN-3110 was administered intra-tumorally in a 3 + 3 dose-escalation design (NCT03152318) [122]. Importantly, this study aimed to collect paired tumor samples (pre- and post-treatment) to inform longitudinal tissue analysis of treatment response. Biopsy and/or resection was obtained for the majority of patients at progression or post-mortem. The treatment was safe and well-tolerated, with no occurrences of dose-limiting toxicity. Additionally, from histologic, immunohistochemical, and quantitative analyses of tissue samples, significant increases in CD8+ and CD4+ T cells were observed in most paired samples following treatment. In HSV-1 seropositive patients with recurrent GBM, post-treatment T cell increases were significantly correlated with post-treatment survival (*p* = 0.017 (CD8+), *p* = 0.026 (CD4+)) [122]. This study demonstrates the role of serial tissue collection and analysis in assessing cellular-level responses to treatment. These responses can help characterize mechanisms of treatment efficacy, as evidenced by their significant association with overall outcomes in a subset of patients. A deeper understanding of the tissue-level changes that occur in response to treatments will be critical to refining the design of future therapies.

### 5.3. Adenoviruses

Aglatimagene besadenovec (AdV-tk) is a non-replicating adenovirus with the *herpes simplex virus (HSV) thymidine kinase (tk)* gene. After this vaccine is delivered locally, patients receive herpes antiviral drugs that are converted to acyclovir and modified further at cells that express HSV thymidine kinase, resulting in the generation of a toxic nucleotide [123,124]. Actively dividing cells are particularly susceptible to this approach. AdV-tk has been studied in several tumor types, and multiple trials in gliomas have been performed [118].

A Phase 1b study (NCT00751270) for patients with high-grade gliomas (10 GBM, 2 anaplastic astrocytoma) administered AdV-tk followed by valacyclovir [125]. Patients received local delivery of AdV-tk immediately following surgery, then received valacyclovir for 14 days. Radiation therapy started within a week of surgery, and temozolomide was administered following completion of acyclovir. No dose-limiting toxicities were observed, and median post-therapy survival for GBM patients was 10.9 months [125]. A Phase 2a study (NCT00589875) for patients with high-grade glioma similarly studied administration of AdV-tk followed by valacyclovir alongside standard of care [126]. This study included patients from NCT00751270 as well as an additional 36 patients (34 GBM, 1 anaplastic astrocytoma, 1 oligodendroglioma). Again, no dose-limiting toxicities occurred, and median post-treatment survival for all GBM patients was 16.7 months [126].

A Phase 2 study (NCT00870181) for adult recurrent high-grade glioma studied AdV-tk followed by ganciclovir [127]. Patients received cerebral intra-arterial infusion of AdV-tK followed by systemic ganciclovir for 14 days. Mannitol was administered prior to treatment as a method to enhance blood–brain barrier disruption. Each cycle lasted 21 days, and patients received at least 2 cycles. Treatment was overall well-tolerated with safety comparable to other treatments for high-grade glioma. mOS for the 14 GBM patients included in the trial was 10.4 months [127].

A Phase 1 trial (NCT03072134) of patients with newly diagnosed malignant glioma studied the conditionally replicative adenovirus CRAd-Survivin-pk7, which contains a tumor-specific human survivin promoter [128]. This product was also modified for improved binding to heparan sulfate proteoglycans that are overexpressed in glioma. Due to their ability to bypass the blood–brain barrier and their affinity for central nervous system malignancy [129,130], neural stem cells were selected as the vehicle by which to deliver the product (NSC-CRAd-S-pk7). Twelve patients were enrolled in this trial, of which eleven had GBM. The product was administered in combination with standard of care radiation and chemotherapy. No dose-limiting toxicities were observed. mOS for all patients was 18.4 months, and mPFS was 9.1 months [128]. An ongoing Phase 1 clinical trial (NCT05139056) is evaluating NSC-CRAd-S-pk7 in patients with recurrent high-grade glioma. Patients undergo an initial surgery, followed by intracerebral administration of up to four weekly doses of the product administered initially manually and subsequently via Rickham catheters, followed by a second surgery two weeks after the last dose of the product to for catheter retrieval and post-treatment tissue sampling. Trial results are pending at this time [131].

The conditionally replicative adenovirus DNX-2401 was engineered to target cells with altered retinoblastoma pathways. In a Phase 1 clinical trial for recurrent GBM, local delivery of DNX-2401 via CED was found to be safe, with promising clinical responses [84]. Adenoviruses have been studied as part of combination regimens as well. Combination therapy of DNX-2401 with temozolomide for recurrent GBM was found to be safe and well-tolerated and displayed therapeutic activity [85,132]. In a Phase 1b trial, DNX-2401 in combination with interferon gamma (IFN) was performed for GBM at first or second recurrence, in an effort to enhance immune activation. While a single dose of DNX-2401 was well-tolerated as monotherapy and was associated with clinical activity, the addition of IFN was not well-tolerated, and not associated with a survival benefit [86]. In a Phase 1/2 trial, DNX-2401 administered intra-tumorally in combination with intravenous pembrolizumab for recurrent glioblastoma was likewise found to be well-tolerated. Though the primary efficacy endpoint was not met (objective response rate 10.4%, which was not statistically greater than the prespecified endpoint of 5%), the secondary endpoint of overall survival at 12 months was 52.7%, statistically greater than the prespecified control rate of 20%. 56.2% of patients had stable disease or better, with three patients experiencing durable responses (alive at 45, 38, and 60 months), an exceptional survival benefit in recurrent GBM [87]. This was noted to be especially important given that prior durable responses to immunotherapies had been limited to patients with advantageous biological features [133].

A Phase 1 trial of mesenchymal-derived stem cells loaded with DNX-2401 administered to patients with GBM, IDH-mutant astrocytoma grade 4, gliosarcoma, or wild-type IDH-1 anaplastic astrocytoma is currently ongoing (NCT03896568). The design of this trial includes an innovative intra-arterial delivery method. A transfemoral approach is used for endovascular super-selective intra-arterial (ESIA) infusions of stem cells. A fusion of MRI and cone beam CT enables the selection of optimal vessels and confirmation of treatment delivery. Combining these radiographic and delivery techniques produces perfusion-guided ESIA (PG-ESIA), which facilitates therapeutic administration via super-selective intracranial delivery and tumor targeting [134].

### 5.4. Reoviruses

Another class of viral vaccines was derived from reoviruses, double-stranded RNA viruses that display preferential replicative potential and toxicity in transformed cell lines [135,136]. A product derived from reoviruses, Reolysin, was evaluated in a Phase 1 trial for recurrent malignant gliomas (anaplastic astrocytoma, *n* = 3 and GBM, *n* = 12). Patients received a single intratumoral stereotactic injection of Reolysin across a range of dose levels. The drug was found to be overall well-tolerated. mOS was 140 days. During the study period of 24 months, ten patients had a stable disease as their best response, one patient had PR, and four had progressive disease. One patient with grade 4 disease survived nearly 3 years post-virus administration, while another with grade 4 disease survived nearly 2 years. It should be noted that IDH mutation status was not reported within the initial data of this study, published in 2014. This study was also the first to use convection-enhanced delivery (CED) for the application of viral delivery to brain tumors [88].

An additional Phase 1 study of Reolysin in combination with GM-CSF and standard of care chemoradiotherapy for newly diagnosed GBM was completed in 2019 in the UK. Across two dose levels, the regimen was overall well-tolerated, with a mOS of 13.1 months was observed, with 12.6 months in the lower dose group and 16.1 months in the higher dose group [137]. The outcome particularly in the higher dose group was promising, with a slight survival benefit over that observed from current standard of care as reported by Stupp et al. [2].

In a window-of-opportunity trial of high-grade gliomas and brain metastases, intravenous infusion with *Orthoreovirus* led to infection of tumor cells, increased cytotoxic T cell infiltration relative to patients who were not infused with the virus. Importantly, the treatment also resulted in upregulation of the PD1/PD-L1 pathway in tumors via an interferon-mediated mechanism, raising an opportunity for synergistic treatment with checkpoint blockade. Accordingly, the authors also performed a preclinical study in which murine models implanted with glioma cells were found to have improved survival after GM-CSF/reovirus treatment followed by PD-1 antibody treatment, compared to either viral therapy or checkpoint blockade alone [138].

### 5.5. Murine Leukemia Virus

Vocimagene amiretrorepvec, also known as Toca 511, is a non-lytic murine leukemia retrovirus that selectively targets dividing cells [139]. The virus was modified to contain a cytosine deaminase capable of converting the pro-drug 5-fluorocytosine (5-FC) to the chemotherapeutic 5-fluorouracil (5-FU). Thus, cellular transfection with Toca 511 and treatment with 5-FC would be expected to yield a specific intra-tumoral chemotherapeutic exposure [140,141]. In a Phase 1, first-in-human study (NCT01156584), 36 patients with recurrent malignant glioma received monthly 5-FC following Toca 511 treatment [142]. Toca 511 was administered either via stereotactic transcranial injection into the tumor, or given as an intravenous injection, depending on cohort. This regimen was well-tolerated, without any dose-limiting toxicities [142]. A subsequent Phase 1 clinical trial (NCT01470794) administered Toca 511 in escalating doses to patients with recurrent high-grade glioma, followed by oral FC administration 6 weeks post-surgery. Median survival was 11.9 months [143]. Finally, in a Phase 2/3 clinical trial for patients with recurrent GBM or anaplastic astrocytoma, 403 patients were randomized to receive either standard of care or Toca 511 with FC (NCT02414165). A total of 201 patients were randomized to the treatment arm. However, no difference in survival was observed, with mOS 11.1 months for the study arm and 12.2 months for the standard of care arm, and the study was ultimately terminated for futility [144]. Subsequent analysis of this study suggested that inadequate dosing of the prodrug likely posed a significant issue, as patients on the trial received a median of two cycles of the prodrug, whereas three–four cycles were required for optimal antitumor response in preclinical models [145]. A Phase 1b clinical trial (NCT02598011) and a Phase 2/3 trial (NCT04105374) evaluating Toca 511 with FC compared to standard of care have since been withdrawn.

### 5.6. Poliovirus

Poliovirus has been studied with some promising results in the treatment of GBM. The poliovirus receptor CD155 is known to exhibit strong expression in solid tumors including GBM and in the tumor microenvironment, supporting the use of a poliovirus vaccine in this setting. In a Phase 1 clinical trial, convection-enhanced, intratumoral delivery of a chimeric, non-pathogenic polio-rhinovirus (PVSRIPO) was evaluated in patients with recurrent GBM [146]. Among 61 patients who received a dose of PVSRIPO, mOS was 12.5 months, longer than the mOS of 11.3 months observed in the historical control group. Moreover, patients who received PVSRIPO reached a plateau in survival. Beginning at 24 months, overall survival in patients who received PVSRIPO was 21% at both 24 and 36 months, compared to 14% and 4%, respectively, in the historical control group [146]. Though this study was performed prior to revision of GBM diagnostic criteria to exclude patients with mutations in IDH, a subset analysis showed that survival among patients without the IDH R132H mutation was equivalent to the survival for all patients (IDH-mutant and wild-type) who received the treatment (12.5 months in both groups). The 21% survival rate at 21 and 36 months had included 45 patients without the IDH R132H mutation [146]. Recently, studies evaluating PVSRIPO as monotherapy (NCT02986178) or in combination with pembrolizumab (NCT04479241) were completed in early 2024, though results are unavailable at the time of this publication. A study evaluating PVSRIPO in combination with atezolizumab was withdrawn (NCT03973879) [111,147].

### 5.7. Measles

Attenuated strains of the measles virus (MV) have been considered attractive options for OV treatment given their minimal safety risk at both the patient and population levels [148,149], tumor tropism, and ability to be genetically engineered [150].

Preclinical studies of MV treatment for GBM have shown promising results, especially in combination with checkpoint inhibitors. In a preclinical study, a murine orthotopic GBM model was treated with either MV infection, an anti-PD-1 agent, or a combination. MV infection was found to upregulate PD-L1 in vitro, and combination treatment synergistically improved survival [151]. A subsequent preclinical study used a Helicobacter pylori-derived protein as an immunostimulatory agent expressed in an MV vehicle; when combined with anti-PD1 treatment in murine models, a synergistic effect was observed with up to 80% cure rate [152].

In a first-in-human Phase 1 clinical trial, 22 patients with recurrent GBM received a carcinoembryonic antigen-expressing oncolytic MV derivative either at the resection cavity, or initially intratumorally and subsequently in the resection cavity following resection [153]. The treatment was well-tolerated, and no dose-limiting toxicities were observed. mOS in this early-phase trial was 11.6 months and one year survival was 45.5% [153]. Additionally, noting that preliminary results of a prior Phase 1 study identified constitutive activation of the interferon pathway as an important predictor for MV replication [154], the present study also developed a model for selecting patients with the most potential sensitivity to the study treatment based on relevant signatures of genetic expression. In a post hoc analysis, a 22 interferon stimulated gene (ISG) diagonal linear discriminate analysis (DLDA) classification algorithm was inversely correlated with viral replication and immunogenic microenvironment remodeling [153]. Altogether, the results of this study support further investigation of therapeutic MVs in this setting and demonstrate an important application for genetic-based predictive modeling in drug development and personalized treatment.

## 6. Vaccine Therapy

The principle of vaccine therapy is sensitization of the host immune system to antigens strongly and specifically expressed in tumors, thus targeting tumor cells while sparing normal cells. This process involves immune cell priming and recruitment [155]. Vaccine therapies for high-grade gliomas are discussed in detail below and summarized in Table 3.

### 6.1. Dendritic Cell Vaccines

Noting the natural role of dendritic cells in antigen presentation for immune cell activation, vaccines that enhance dendritic cell ability were developed. Dendritic cells (DC) may be designed to target a range of moieties, including a spectrum of antigens in autologous tumor lysate, nucleic acids, and peptides [111]. The result is stimulation of both CD4+ and CD8+ T cell populations against the inoculated entity.

Perhaps the most notable DC vaccine is DCVax-L, which was developed for reactivity against autologous tumor lysate. This strategy offered a personalized approach to treatment that would account for the intra-tumoral heterogeneity intrinsic to a patient’s own tumor. An international, multicenter trial 94 sites across four countries) enrolled 331 patients with newly diagnosed GBM. In this nonrandomized, externally controlled trial, DCVax-L was associated with increased mOS in both newly diagnosed GBM (19.3 months DCVax-L vs. 16.5 months for external controls) and recurrent GBM (13.2 months DCVax-L vs. 7.8 months external controls) [73]. However, several limitations must be considered when assessing the significance of these results. The design of the trial was a crossover study in which all patients who received placebo had the option to cross over to receiving DCVax-L on recurrence, and a high rate of ensuing crossover (90%) was observed. An external control cohort was therefore designated. Additionally, the primary outcome was changed during the trial. The original primary endpoint of radiographically determined PFS was later changed to OS due to the difficulty of discerning true disease progression from pseudoprogression. Finally, the vaccine administered to patients upon recurrence was derived from their initial tumor sample at the time of diagnosis, though extensive evidence demonstrates the tendency of GBM undergo molecular transformation from initial stage to recurrence [156,157,158].

Novel DC vaccine manufacturing techniques have been developed to overcome limitations from manufacturing yield. One such approach used a platelet lysate-based supplement to enhance growth and stem-like marker expression of human GBM cell lines, and a modified culturing method that produced high levels of mature DCs compared to standard culturing methods [159]. Subsequently, a Phase 1 trial assessed this optimized DC product in the treatment of patients with newly diagnosed GBM [160]. Following standard of care surgery and chemoradiation, patients received intradermal DC vaccines and temozolomide. Safety was confirmed, with no dose-limiting toxicities. mOS was 19 months and mPFS was 9.7 months, with one patient remaining progression-free for 5 years after enrollment [160]. Continued manufacturing advancements and evaluation in later phase clinical trials will be important for optimizing efficacy and expanding applicability of these products.

### 6.2. Peptide Vaccines

Peptide vaccines are designed with protein sequences that distinguish a tumor (tumor-specific antigens or tumor-associated antigens). When these vaccines are administered, the peptides are presented by the major histocompatibility complex (MHC), activating the cellular immune response [161]. As the humoral immune response is not readily activated through this method, peptide vaccines are often combined with other immunotherapies [161]. Several proteins have been found to be frequently mutated in GBM, though only a subset of them have been developed into peptide vaccines due to low rates of conservation across the GBM population [162]. Among these are EGFRvIII, survivin, CMV antigens, and multi-peptide or individualized vaccines.

EGFRvIII is estimated to occur in 20–30% of all patients with GBM, and in 50–60% of GBM patients with EGFR amplification [163]. In a Phase 2, multicenter clinical trial, the experimental EGFRvIII-targeting vaccine Rindopepimut was administered intradermally to patients receiving standard dose or dose-intensified (DI) temozolomide. Results from this trial were promising, with EGFRvIII-specific immune responses effected in all patients, and EGFR-expressing tumor cells eradicated in almost all patients. mPFS and mOS exceeded those of historical controls [164]. However, a subsequent Phase 3 clinical trial evaluated Rindopepimut in patients with newly diagnosed GBM with EGFRvIII amplification who had received standard of care chemoradiation. Compared to standard of care with placebo, standard of care with Rindopepimut did not show a survival benefit [58]. An important unexpected result discovered in this study was the loss of EGFRvIII in 60% of the small sample of patients for whom tissue was available at recurrence, regardless of whether they received the study treatment. From the lack of significant benefit associated with Rindopepimut to the potential instability of its sole target peptide EGFRvIII, this study ultimately emphasized the need for evaluating vaccines directed against several targets or capable of inducing epitope spreading through widespread immunoactivity in order to improve therapeutic breadth. This study also highlighted the importance of biopsy confirmation of EGFRvIII positivity in studies examining this mutation in trials of patients with recurrent GBM [58].

The anti-apoptotic protein survivin (BIRC5) is strongly expressed in most tumors. A study of quantitative expression levels showed that survivin was expressed in 80% of GBM samples [165]. Though overexpressed during embryonic development, survivin is not present in differentiated tissues [166]. Survivin was thus identified as a promising therapeutic target. In a Phase 2a, open-label, multicenter trial, the survivin-targeted peptide vaccine SurVaxM was administered to patients with newly diagnosed GBM in combination with adjuvant temozolomide following surgery and radiation. Among 64 patients who were enrolled and received SurVaxM, treatment was safe and well-tolerated, and 60 of the 63 patients remained progression-free 6 months after diagnosis, with mPFS 11.4 months and mOS 25.9 months [167]. A Phase 2b randomized controlled trial has since been launched to further investigate safety and efficacy, with a total planned enrolment of 228 patients of which 137 will receive the treatment [168]. This trial (SURVIVE, NCT05163080) is currently active.

Cytomegalovirus (CMV) antigens represent promising targets for GBM therapy, given that CMV proteins have been identified within most GBMs, but not in normal brain tissue [169]. In particular, the CMV phosphoprotein 65 (pp65) has been the focus of several prior trials. A single-arm, Phase 1 trial of 11 patients with newly diagnosed GBM evaluated a CMV pp65 vaccine alongside standard of care (NCT00639639). This trial demonstrated significant benefits in mPFS (25.3 months, 95% CI 11–∞) and mOS (41.1 months, 95% CI 21.6–∞) compared to matched historical controls, and four patients remained progression-free at 59 to 64 months from diagnosis [170]. In a Phase 1 open-label, first-in-human trial CMV-specific adoptive cellular therapy (ACT) was administered to 25 patients with primary GBM. No patients experienced toxicity (ACTRN12615000656538). Long-term follow up revealed significantly improved OS compared to patients who progressed prior to ACT (mOS 23 months vs. 14 months, *p* = 0.018) [171]. More recently, a first-in-human Phase 1 trial (NCT03299309) assessed a pp65-targeted peptide vaccine (PEP-CMV) in children and young adults with recurrent high-grade glioma and medulloblastoma [172]. The treatment was overall well-tolerated and associated with increased T cell reactivity, serving as evidence of the treatment’s ability to induce an immune response even in patients who had received multiple prior treatments and experienced multiple recurrences. mPFS was 2.5 months and mOS was 6.4 months; 12-month OS was 26.6% [172]. Based on these data, a multi-institutional Phase 2 trial evaluating PEP-CMV for pediatric recurrent medulloblastoma, newly diagnosed HGG, and newly diagnosed DIPG is currently open and recruiting (NCT05096481).

A multipeptide vaccine, IMA950, has also been developed. This vaccine is composed of 11 tumor-associated antigens, including nine MHC class I-restricted peptides and two MHC class II-restricted peptides. Specificity of the MHC class I peptides is conferred by high expression on GBM tumor cells, but absent expression in normal cells [173]; the MHC class II peptides have not been isolated from the surface of GBM samples, but prior vaccine trials have demonstrated their immunogenicity [174,175,176,177,178]. A Phase 1/2 clinical trial (NCT01920191) studied IMA950 combined with adjuvant poly-ICLC [178], an immunostimulatory double-stranded RNA molecule that exerts downstream activity in a manner mimicking a viral infection [179]. Treatment was administered to 16 patients with newly diagnosed GBM and was largely safe, though 4 patients developed short-term cerebral edema. Immunogenicity was observed, with a mOS of 19 months. In a post hoc exploratory study, patients on this trial received bevacizumab upon recurrence to identify a potential combinatorial benefit between prior IMA950/poly-ICLC vaccination and bevacizumab, though there was no difference in mPFS (2.6 vs. 4.2 months for vaccinated vs. control patients, *p* = 0.5) or mOS (7.8 vs. 10.0 months for vaccinated vs. control patients, *p* = 0.69) [180]. Other combination regimens including IMA950/poly-ICLC have been investigated as well, including a Phase 1/2 trial (NCT03665545) investigating IMA950 and poly-ICLC in combination with pembrolizumab that was reported in 2020 [181]. Preliminary results showed an increase in peripheral CD4 and CD8 counts in response to vaccination [181], however updated data from this trial is not available. Additionally, while outside the scope of this review focusing on immunotherapies for high-grade gliomas, a recent pilot study evaluating the safety and immunogenicity of IMA950/poly-ICLC combined with the agonistic anti-CD27 antibody varlilumab in low-grade gliomas is worth noting. Among 10 patients with low-grade gliomas who received the combination regimen, treatment was well-tolerated and associated with reactive T cell expansion in the peripheral blood, but a detectable response in the tumor was not achieved [182].

Another multipeptide vaccine, EO2401, was studied in progressive GBM in a Phase 1/2 study (NCT04116658). This microbiomial-derived product contained synthetic HLA-A2 restricted peptides mimicking antigens overexpressed in GBM (IL13Rα2, BIRC5 and FOXM1), and the CD4 helper peptide UCP2 [183]. At first progression of GBM, patients received either the vaccine alone, in combination with nivolumab, or in combination with nivolumab and bevacizumab). Strong peptide-directed T cell responses were attained in the majority of patients, indicating immunogenicity. Among patients who received EO2401 + nivolumab, mPFS was 1.8 months; patients who additionally received bevacizumab experienced extended mPFS to 5.5 months [183].

Personalized vaccine therapies have also garnered interest for their potential to offer an individual, tumor-specific approach to treatment. In a Phase 1 trial of the Glioma Actively Personalized Vaccine Consortium (GAPVAC-101, NCT02149225), 15 patients with newly diagnosed GBM were administered a series of two vaccines: APVAC1 (derived from a library of unmutated tumor antigens), then APVAC2 (derived from tumor-specific neoepitopes) [184]. Treatment generated immunogenicity, with sustained responses of CD8+ cells and CD4+ cells to APVAC1 and APVAC2, respectively. Treatment was overall well-tolerated (three serious adverse events were documented). mOS was 29.0 months from diagnosis, and mPFS was 14.2 months [184]. A Phase 1 trial assessing another personalized neoantigen vaccine with radiation therapy and pembrolizumab in patients with newly diagnosed GBM is currently underway (NCT02287428). An autologous, formalin-fixed tumor vaccine (AFTV) developed from resected GBM was studied in a Phase 2b trial of patients with newly diagnosed GBM, who received the vaccine intradermally over three courses before and after chemoradiation. While no significant difference in OS or PFS was identified between patients who received the AFTV and placebo overall, a significant benefit was identified among patients who had undergone a total tumor resection prior to receiving the AFTV (3-year OS: treatment 80%, placebo 54%, *p* = 0.16; 3-year PFS: treatment 81%, placebo 46%, *p* = 0.0067) [185]. These results led to the development of an ongoing Phase 3 randomized controlled trial evaluating the AFTV in patients with newly diagnosed GBM who had previously undergone a gross total resection [186]. Thus far, Phase 3 trials for personalized peptide vaccines have overall been limited in number and in ability to demonstrate therapeutic benefit. A personalized peptide vaccine for HLA A-24+ recurrent GBM was studied in a Phase 3 trial of 88 patients, but failed to meet either the primary endpoint of OS, or any of various clinical and biologic secondary endpoints [187].

### 6.3. Nucleic Acid Vaccines

Nucleic acid-based vaccines include DNA in the form of plasmids, or RNA in the form of mRNA as a substrate for translation. These vaccines have important implications in the treatment of both infectious disease and cancer. Among the many advantages of DNA vaccines include efficacy (via activation of both cellular and humoral immunity) [188,189], safety as demonstrated in both preclinical and clinical trials of various solid tumors [190,191], and the ability to administer repeat infusions due to the absence of anti-DNA antibody formation [192]. Additionally, the CpG component of plasmid vectors may inhibit the activity of regulatory T cells (Tregs), which otherwise proliferate and differentiate in response to tumor growth, leading to immune suppression [189,193,194,195].

A DNA plasmid-based drug was evaluated in progressive GBM, with promising results. VXM01 is composed of an attenuated *Salmonella typhi* strain harboring a DNA plasmid encoding vascular endothelial growth factor receptor (VEGFR-2). Administration of VXM01 invokes a systemic T cell response against VEGFR-2, an important factor in tumor neoangiogenesis. In a Phase 1 clinical trial, 14 patients with progressive GBM received VXM01 via oral administration; 3 patients also received nivolumab, and 8 patients also underwent surgery. VXM01 was found to be safe, and associated with a measurable immune response as reflected by a post-administration increase in the CD8/Treg ratio [196]. Importantly, patients with longer survival were found to have decreased tumoral PD-L1, suggesting a role for combination treatment of VXM01 with an anti-PDL1 agent. VXM01 in combination with avelumab was thus studied in a Phase 1/2 clinical trial of 28 patients with recurrent GBM. Among non-resectable patients, three partial responses were observed, two of whom were progression-free for over 12 months. In one resected patient, survival exceeded 18 months [197].

mRNA vaccines likewise have a strong safety profile due in part to degradations precluding genome integration [198] and low intrinsic immunogenicity [189]. At the cellular level, cytoplasmic translation of the mRNA bypasses the barrier of nuclear penetration [189]. A preclinical study of personalized, whole transcriptome-derived tumor RNA packaged into lipid nanoparticles showed safety and activity in murine models and in a canine. Cationic modification of the nanoparticle facilitated lymphoid localization and in turn augmented the peripheral immune response, contrasting with previous anionic nanoparticle products which had localized to the spleen [91,92]. Increased intra-tumoral immunogenicity and sensitization of a ‘cold’ tumor environment to checkpoint inhibitor therapy were also achieved. Translationally, a personalized mRNA nanoparticle was then administered to a canine with spontaneous malignant gliomas and demonstrated activity and tolerability [91,92,93]. In 2013, an mRNA vaccine therapy was studied in an early phase clinical study of seven patients with GBM who received a vaccine of dendritic cells transfected with mRNA from autologous cancer stem cells. mPFS of vaccine recipients in this study was 2.9 times longer than in matched controls (median 694 vs. 236 days) [199]. However, these results should be interpreted in the context of the small sample size and short survival time of the control cohort, which together limit accurate analysis of comparative benefit. Noting the significant presence and immunogenicity of the Wilms’ tumor protein across a range of malignancies [200], mRNA encoding the Wilms’ tumor protein was developed into an autologous dendritic cell vaccine (WT1-mRNA/DC) and has been studied in a single-arm, Phase 1/2 trial of patients with advanced solid cancers who were receiving standard therapy, including 13 patients with GBM [201]. The treatment was well-tolerated and suggested a potential survival benefit in this early stage, with a mOS of 43.7 months across the 13 patients with GBM [201]. Larger sample sizes, later phase clinical trials, the use of a randomized controlled design, and subgroup analyses to identify patient populations and molecular features conferring sensitivity to this treatment will be important variables to consider as these results are validated in subsequent trials.

### 6.4. Heat Shock Proteins

Heat shock proteins (HSPs) function as intracellular peptide chaperones. HSPs serve a critical function in antigen presentation for immune stimulation [202]. Autologous HSPs derived from a patient’s tumor may thus be developed into polyvalent vaccines. Prior Phase 1 and 2 single-arm trials in recurrent GBM demonstrated safety, robust immune stimulation, and a modest survival benefit in response to treatment with an HSP vaccine [203,204]. Based on these data, a single-arm, Phase 2 study evaluated the addition of an autologous HSP vaccine to standard of care surgery and chemoradiation for newly diagnosed GBM. Among 46 patients who received this regimen, mOS was 23.8 months [205]. mOS was significantly higher for patients with low peripheral myeloid PD-L1 expression (44.7 months) compared to patients with high PD-L1 expression (18.0 months), indicating the importance of PD-L1 expression in immunosuppression and vaccine efficacy [205]. Additionally, a single-arm Phase 2 study evaluated an autologous HSP vaccine for recurrent GBM, finding that 90.2% of the 41 patients treated were alive at 6 months, and mOS was 42.6 weeks [203]. Patients with lymphocyte counts below the cohort median exhibited decreased OS (*p* = 0.012). Further investigation of HSP vaccines in later phase, randomized controlled trials for GBM will be important to determine treatment efficacy at different timepoints in the clinical course. Continued consideration and characterization of peripheral immune factors impacting treatment efficacy will be a key component of these analyses.

## 7. CAR-T

Noting the importance of the immune system in combatting cancer, chimeric antigen receptor (CAR) T- cell therapy was developed. CAR-T is a form of adoptive cell therapy, in which T cells are engineered to express an extracellular high-affinity receptor for tumor antigens and an intracellular T cell costimulatory domain, resulting in enhanced antitumor immunogenicity [206,207]. Through this process, T lymphocytes are isolated from a patient and altered in vitro. The patient then undergoes lymphodepletive chemotherapy prior to infusion of the CAR-T product. CAR-T has achieved exceptional success in the treatment of hematological malignancies [208]. However, results in the treatment of solid malignancies, including GBM, have been more limited. Antigens previously studied in CAR-T for high-grade gliomas include IL-13Rα2, EGFRvIII, and GD2. Relevant clinical trials are discussed in detail below and summarized in Table 4.

### 7.1. IL-13Rα2

IL-13Rα2 was considered an attractive target for CAR-T after early studies showed that the majority of GBM samples frequently overexpressed its receptor, with greater specificity and homogeneity than other growth factor receptors [209,210]. After a case of durable response to IL-13Rα2- targeting CAR-T in relapsed multifocal GBM was reported in 2009 [211], a Phase 1 clinical trial was performed to evaluate safety and tolerability of a CAR-T product against IL-13Rα2 in recurrent high grade glioma, the majority of which was GBM. [62] Treatment was administered via three routes: intra-tumoral, intraventricular, and a dual intra-tumoral/intraventricular route. The treatments were safe, well-tolerated, and associated with increased inflammatory cytokines in the central nervous system, correspondent with CAR-T bioactivity. Among recurrent GBM patients, the dual delivery route was associated with mOS 10.2 months compared to 7.7 months for all patients with recurrent GBM [62]. Later phase studies will be needed for further evaluation of efficacy.

### 7.2. EGFRvIII

Epidermal growth factor receptor variant III (EGFRvIII) is a in which exons 2–7 are deleted, producing a constitutively activated receptor. EGFRvIII occurs in up to 30% of high-grade gliomas, especially GBM, and is correlated with poor prognosis [212]. Given its relatively high incidence and prognostic significance, EGFRvIII was considered a promising CAR-T target. In a first-in-human Phase 1 study of intravenous administration of a single dose of CAR-T directed against EGFRvIII in recurrent GBM, the treatment was safe, tolerable, and associated with decreased antigen expression and increased regulatory T cell infiltration in the tumor microenvironment. This study was largely observational, though mOS among the ten patients studied was limited at 251 days [59]. Among the proposed obstacles to efficacy were the immunosuppressive nature of the tumor microenvironment, which was amplified after CAR-T infusion, and the intra-tumoral heterogeneity of EGFRvIII expression. In contrast to normal brain, recurrent tumor cells also expressed wild-type EGFR.

A secreting CAR T product against EGFR was subsequently developed to target both EGFRvIII and wild-type EGFR [213]. The wild-type EGFR protein was targeted through secretion of a T-cell-engaging antibody molecule (TEAM). In a first-in-human Phase 1 clinical trial, the Intraventricular CARv3-TEAM-E T Cells in Patients with Glioblastoma (INCIPIENT) study, the safety of CARv3-TEAM-E T cells was evaluated in patients with both newly diagnosed and recurrent GBM [213]. Thus far, the results of the first three patients treated have been published. No dose-limiting toxicities or adverse events greater than grade 3 occurred. Though radiographic improvement within a day of intraventricular infusion was observed, this response was transient in two of the three patients studied (with recurrence within 1–3 months) [213]. The data from this trial should be interpreted with caution, noting the very small sample size. Still, the failure to show a reliably durable response emphasizes the need for enduring treatments that extend the time to recurrence by a clinically significant interval.

### 7.3. Bivalent Therapy

Recognizing the role of intratumoral heterogeneity in limiting prior CAR T efficacy [59], a Phase 1 trial studied intrathecally delivered bivalent CAR T cells targeting both EGFR and IL13Rα2 in 6 patients with recurrent GBM [63]. Two dose levels were administered. At both dose levels, patients experienced early-onset neurotoxicity that was managed with dexamethasone and anakinra, and one patient in dose level 2 experienced dose-limiting toxicity. While decreased enhancement in tumor size was observed in all six patients, none met criteria for objective radiographic response, despite all patients receiving high dose dexamethasone or bevacizumab within the first month following therapy. Exploratory endpoint analysis showed high levels of CAR T cells and cytokine release in the CSF of all six patients. Though the treatment was safe in a majority of patients and displayed evidence suggesting bioactivity, future trials in subsequent phases are needed to confirm safety and investigate efficacy.

### 7.4. GD2

Diffuse midline gliomas have historically posed a therapeutic challenge due to their treacherous midline location and aggressive clinical course. A breakthrough in the application of CAR-T for diffuse intrinsic pontine glioma (DIPG) and other H3K27-mutated diffuse midline gliomas was fueled by the discovery that the disialoganglioside GD2 was highly and uniformly expressed on these tumors. Preclinical studies showing efficacy of GD2-directed CAR-T against these tumors supported the role for clinical trials in this setting [214,215]. The Arm A results of a Phase 1 clinical trial evaluating GD2 CAR-T for H3K27-mutant pontine gliomas (DIPG) or spinal diffuse midline gliomas (sDMG) were recently published [216]. In this arm of a first-in-human trial, nine DIPG and two sDMG patients were administered an intravenous dose of autologous GD2 CAR-T at one of two dose levels following lymphodepleting chemotherapy. Those with clinical or radiographic benefits were then eligible for intracerebroventricular (ICV) infusions. Repeat dosing was permittable for patients with radiographic response or stability, clinical benefit, and tolerable safety, among other criteria. All nine patients received ICV without dose-limiting toxicity, though developed tumor inflammation-associated neurotoxicity that was treatable. Ultimately, four patients exhibited major volumetric reductions in their tumor (>50%), and smaller reductions were noted in three additional patients. One patient had a complete response for over 30 months from enrollment, and all nine patients experienced neurological benefit [216]. These results mark a promising therapeutic advance for an aggressive tumor entity, for which prior treatments have not reliably been associated with durable response. Future studies in Phase 2 and beyond will be important to further evaluate these results.

## 8. Targeted Cytokines

As a key modulator of the immune response, cytokines have been studied for their role in cancer treatment. Cytokines may serve either a pro- or an anti-inflammatory function [217]. While their role in immune modulation suggests a benefit to targeting them for anticancer benefit, this must be balanced against the potential for off-target side effects [218]. Additionally, due in large part to their short half-life and narrow therapeutic window, the promising results observed in the preclinical setting have largely failed to translate into clinical trial efficacy [219]. To date, only two cytokines, IL-2 and IFN-α, have been FDA approved for the treatment of cancer. IL-2 has frequently been used in CAR T and adoptive T cell therapy trials [220,221,222]. However, neither agent has received approval as a monotherapy for tumors of the CNS. Clinical trials evaluating cytokine therapies for high-grade gliomas are described below and summarized in Table 5.

To expand the efficacy of cytokines, recent efforts have focused on combinatorial strategies. A prior Phase 3 clinical trial of 199 patients with high-grade gliomas administered standard of care radiation + temozolomide to all patients, followed by randomization to receive either temozolomide alone or temozolomide + IFN- α. mOS of patients who received temozolomide + IFN-α was 26.7 months, compared to 18.8 months in the standard of care group (*p* = 0.005) [223]. However, a separate Phase 3 clinical trial of high-grade glioma patients showed that IFN-α did not improve OS or time to recurrence when added to radiation therapy and carmustine [224]. As several prior trials of vaccines in GBM have largely been unsuccessful in meaningfully improving survival as detailed above (see Section 6), more recent strategies have sought to improve outcomes through the addition of cytokines. As described above, the HSV-1 oncolytic therapy M032 was engineered to induce IL-12 expression, and in a Phase 1 trial was well-tolerated with preliminary evidence suggesting a favorable response in some patients [119], though later stage trials will be important for further investigation. Cytokines have also been studied in combination with CAR T. A preclinical study of GD2-CARs modified to contain a constitutively active IL-7 receptor was associated with durable tumor elimination in murine GBM xenografts [225], and another preclinical study showed that GD2-CARs with transgenic IL-15 expression achieved a 50% complete response rate in an intracranial xenograft GBM model [226]. As reported above, IL-13Rα2 has been studied as a CAR-T target (see Section 7.1.) in a recent Phase 1 trial demonstrating safety, and preliminarily suggesting favorable outcomes among patients with recurrent GBM who received treatment via a dual intra-tumoral/intraventricular route [62].

Several ongoing studies for combination treatments with cytokines for GBM are currently underway. L19TNF is a recombinant fusion protein containing a human antibody fragment and TNF, and is designed to target a domain of oncofetal fibronectin which is highly expressed in tumor vasculature [227]. Prior Phase 1 trials in solid tumors, especially sarcomas, demonstrated safety and suggested antitumor efficacy [227]. L19TNF is now being studied in the context of GBM. A current Phase 1 clinical study is evaluating L19TNF in combination with standard of care radiation and chemotherapy for newly diagnosed GBM (NCT04443010), and a separate ongoing Phase 1/2 study is investigating L19TNF in combination with lomustine for patients with GBM at first progression (NCT04573192).

Immune checkpoint inhibitors represent a promising complement for cytokine-based regimens, due to their superior safety profile and their demonstrated efficacy in the treatment of several solid tumors [219,228,229]. A Phase 2b clinical trial previously demonstrated that pembrolizumab in combination with the long-acting IL-7 cytokine molecule NT-I7 improved survival in microsatellite-stable colorectal and pancreatic cancer relative to historical controls (colorectal: mOS 13.2 months treatment vs. 10.8 months historical controls; pancreatic cancer: mOS 11.1 months treatment vs. 6.1 months historical controls) [230]. In orthotopic GBM murine models, NT-I7 produced an immunostimulatory effect with significantly increased CD8 cells and decreased regulatory T cells in tumors, and significantly improved survival [231]. Subsequently, a Phase 1 study showed that in 19 patients with newly diagnosed high-grade gliomas who completed temozolomide and radiation, NT-I7 was well-tolerated [232]. Based on these data, an ongoing Phase 2 study at our home institution is evaluating NT-I7 and pembrolizumab as neoadjuvant and adjuvant therapy with surgery for patients with recurrent GBM (NCT05465954).

## 9. Conclusions

High-grade gliomas, especially GBM, are associated with significant mortality. The development of effective treatments that extend survival is essential and urgent. Immunotherapies have been harnessed for the treatment of high-grade gliomas, owing to the recognized potency of the immune system in targeting and combatting pathogenic states. Checkpoint inhibitors, viruses, vaccines, CAR-T, and targeted cytokines as part of single and multimodal approaches have all been studied for their potential to improve outcomes in high-grade gliomas. However, even where early phase trials have shown promising results, later phase clinical trials have largely failed to demonstrate significant clinical improvements. Now, keen attention to the mechanisms that promote malignant potential, limit immunotherapy efficacy, and drive treatment resistance should be prioritized. As the field evolves, meeting an expanded arsenal of potential treatment options with more comprehensive and dynamic methods of response assessment will become crucial. Critical analysis of prior trials and the reasons they failed, both strategically and mechanistically, will be key to designing future trials with a stronger chance of success. Innovative development of novel therapeutics, thoughtful design of sequential and combination strategies, and algorithm-guided personalized application where possible must convergently be implemented to advance this field in a clinically meaningful direction.

## Figures and Tables

**Table 1:** Active and recently completed clinical trials evaluating immune checkpoint inhibitors for the treatment of high-grade gliomas.

Trial Identifier	Official Study Title	Phase	Additional Interventions	Tumor Types	Current Status
NCT05463848	Surgical Pembro ± Olaparib W TMZ for RGBM	2	Surgery	Recurrent GBM	Recruiting
NCT04977375	Trial of Anti-PD-1 Immunotherapy and Stereotactic Radiation in Patients With Recurrent Glioblastoma	1/2	Surgery	Recurrent GBM	Recruiting
NCT05084430	Pembrolizumab and M032 (NSC 733972) in Treating Patients with Newly Diagnosed or Recurrent/Progressive Glioblastoma, Anaplastic Astrocytoma, or Gliosarcoma	1/2	Surgery	Newly Diagnosed GBM, Recurrent GBM, Anaplastic Astrocytoma, Gliosarcoma	Recruiting
NCT06556563	EF-41/KEYNOTE D58: Phase 3 Study of Optune Concomitant With Temozolomide Plus Pembrolizumab in Newly Diagnosed Glioblastoma	3	-	Newly Diagnosed GBM	Recruiting
NCT02658279	Pembrolizumab (MK-3475) in Patients With Recurrent Malignant Glioma With a Hypermutator Phenotype	2	-	Recurrent Malignant Glioma	Active, Not Recruiting
NCT03576612	GMCI, Nivolumab, and Radiation Therapy in Treating Patients With Newly Diagnosed High-Grade Gliomas	1	AdV-tk (oncolytic virus), valacyclovir (GMCI), SOC	Newly Diagnosed High-Grade Glioma	Completed (results pending)
NCT03277638	Laser Interstitial Thermotherapy (LITT) Combined With Checkpoint Inhibitor for Recurrent GBM (RGBM)	1/2	Stereotactic biopsy	Recurrent GBM	Recruiting
NCT06160206	Retifanlimab With Bevacizumab and Hypofractionated Radiotherapy for the Treatment of Recurrent Glioblastoma	2	-	Recurrent GBM	Recruiting
NCT05465954	Efineptakin alfa (NT-I7) Plus Pembrolizumab for the Treatment of Recurrent Glioblastoma	2	Surgery	Recurrent GBM	Recruiting

Official study title and current status as reported on clinicaltrials.gov; AdV-tk = aglatimagene besadenovec; GMCI = gene-mediated cytotoxic immunotherapy; SOC = standard of care.

**Table 2:** Clinical trials evaluating oncolytic viruses for the treatment of high-grade gliomas.

Trial ID	Virus Species	Product/Strain	Additional Treatments	Phase	Tumor Type(s)	Outcomes
NCT02062827	HSV	M032	-	1	Recurrent/progressive GBM, AA, gliosarcoma	Tolerable safety. Preliminary median post-treatment survival: 9.38 months; trial ongoing.
NCT05084430	HSV	M032	Pembrolizumab	1/2	Recurrent/progressive and newly diagnosed GBM, AA, gliosarcoma	Ongoing
UMIN000002661	HSV	G47Δ	-	1/2	rGBM	Tolerable safety. mOS: 7.3 months, 1-year survival rate: 38.5%. pFS: 8 days from last administration (due to enlargement of enhancing region).
UMIN000015995	HSV	G47Δ	-	2	Residual or rGBM	mOS: 20.2 months. 1-year survival rate: 84.2%.
NCT00751270	Adenovirus	AdV-tk	Valacylovir, SOC	1b	nGBM, nAA	Tolerable safety. Median post-therapy survival: 10.9 months
NCT00589875	Adenovirus	AdV-TK	Valacyclovir, SOC	2a	nGBM, nAA, nAO	Median post-treatment survival for GBM patients: 16.7 months
NCT00870181	Adenovirus	AdV-TK	Ganciclovir, Mannitol	2	rHGG	mOS for GBM patients: 10.4 months
NCT03072134	Adenovirus	NSC-CRAd-S-pk7	SOC	1	nGBM, nAA	Tolerable safety. mPFS for all patients: 9.1 months; mOS: 18.4 months.
NCT05139056	Adenovirus	NSC-CRAd-S-pk7	Surgery	1	rHGG	Ongoing
**	Adenovirus	DNX-2401	-	1	rGBM	Tolerable safety. 4 patients with radiographic response, 1 with complete regression.
**	Adenovirus	DNX-2401	TMZ	1	rGBM (1st recurrence)	Tolerable safety. mPFS: 51 days; mOS: 282 days.
NCT02197169	Adenovirus	DNX-2401	Interferon gamma	1b	rGBM	Addition of interferon not well-tolerated, not associated with survival benefit. DNX-2401 well-tolerated as monotherapy.
NCT02798406	Adenovirus	DNX-2401	Pembrolizumab	1/2	rGBM	Tolerable safety. Primary efficacy endpoint not met (ORR 10.4%, vs. prespecified endpoint of 5%. Secondary endpoint of OS-12: 52.7%, vs. prespecified control rate of 20%. Durable response *n* = 3 (alive at 45, 38, and 60 months).
T03896568	Adenovirus	DNX-2401	-	1	rHGG	Ongoing.
NCT00528684	Reovirus	Reolysin	-	1	rGBM, rAA	Tolerable safety. mOS: 140 days. Note that IDH mutation status was not published within original 2014 data.
CTIS#: 2016-001632-35	Reovirus	Pelareorep	GM-CSF, SOC	1	nGBM	Tolerable safety. mOS: 13.1 months; 12.6 months in the lower dose group, 16.1 months in the higher dose group.
EudraCT: 2011-005635-10	Reovirus	Orthoreovirus	-	1b	rHGGs, brain metastases	Tolerable safety. Median post-treatment survival: 469 days (range 118 to 1079 days)
NCT01156584	Murine Leukemia Virus	Toca 511	5-FC	1	rHGG	Tolerable safety.
NCT01470794	Murine Leukemia Virus	Toca 511	FC	1	rHGG	Tolerable safety. mOS 11.9 months.
NCT02414165	Murine Leukemia Virus	Toca 511	FC	2/3	rGBM, rAA	mOS: 11.1 months for the study arm vs. 12.2 months for the standard of care arm. Study terminated for futility.
NCT01491893	Poliovirus/Rhinovirus	PVSRIPO	-	1	rGBM	Tolerable safety. mOS: 12.5 months, vs. 11.3 months in the historical control group
NCT02986178	Poliovirus/Rhinovirus	PVSRIPO	-	2	Recurrent Grade 4 glioma	Completed early 2024, data unavailable.
NCT04479241	Poliovirus/Rhinovirus	PVSRIPO	Pembrolizumab	2	rGBM	Completed early 2024, data unavailable.
NCT00390299	Measles	MV-CEA	-	1	rGBM	Tolerable safety. mOS: 11.6 months. 1 year survival: 45.5%

** Trial Identification unavailable on ClinicalTrials.gov and CTIS; GBM = glioblastoma; rGBM = recurrent glioblastoma; rHGG = recurrent high-grade glioma; nGBM = newly diagnosed glioblastoma; AA = anaplastic astrocytoma; nAA = newly diagnosed anaplastic astrocytoma; rAA = recurrent anaplastic astrocytoma; nAO = newly diagnosed anaplastic oligodendroglioma; SOC = standard of care; TMZ = temozolomide; FC = flucytosine; mOS: median overall survival; mPFS: median progression-free survival; OS-12: 12-month overall survival; ORR = objective response rate.

**Table 3:** Clinical trials evaluating vaccine therapies for the treatment of high-grade gliomas.

Trial ID	Vaccine Class	Product	Additional Treatments	Phase	Tumor Type(s)	Outcomes
NCT00045968	DC	DCVax-L	SOC (for nGBM)	3	nGBM, rGBM	Newly diagnosed GBM: mOS 19.3 months DCVax-L vs. 16.5 months for external controls; Recurrent GBM: mOS 13.2 months DCVax-L vs. 7.8 months external controls
NCT01957956	DC	Optimized DC vaccine	SOC	1	nGBM	Tolerable safety. mPFS: 9.7 months; mOS: 19 months
**	Peptide: EGFRvIII	Rindopepimut	SOC, with standard or dose-intensified adjuvant TMZ.	2	nGBM	Tolerable safety. mPFS: 15.2 months; mOS: 23.6 months
NCT01480479	Peptide: EGFRvIII	Rindopepimut	SOC	3	nGBM	mOS: 20.1 months rindopepimut, vs. 20.0 months control. Study terminated for futility.
NCT02455557	Peptide: Survivin	SurVaxM	SOC	2a	nGBM	mPFS: 11.4; mOS: 25.9 months
NCT05163080	Peptide: Survivin	SurVaxM	SOC	2b	nGBM	Ongoing.
NCT00639639	Peptide: CMV pp65	CMV-ALT, CMV-DC	SOC +/− GM-CSF	1	nGBM	mPFS: 25.3 months; mOS: 41.1 months
ACTRN126150-00656538	Peptide: CMV pp65	CMV-specific ACT	SOC	1	nGBM	Tolerable safety. mOS: 23 months for treated patients, vs. 14 months for patients who progressed prior to ACT treatment
NCT03299309	Peptide: CMV pp65	PEP-CMV	-	1	rHGG, rMB (children/young adults)	Tolerable safety. mPFS: 2.5 months; mOS: 6.4 months
NCT01920191	Peptide: multipeptide	IMA950	poly-ICLC	1/2	nGBM	Tolerable safety. mOS: 19 months
NCT03665545	Peptide: multipeptide	IMA950	poly-ICLC, Pembrolizumab	1/2	rGBM	Updated data and trial status not available.
NCT04116658	Peptide: multipeptide	EO2401	+/− nivolumab; +/− nivolumab + bevacizumab	1/2	rGBM	Tolerable safety. EO2401 + nivolumab: mPFS 1.8 months; EO2401 + bevacizumab: mPFS 5.5 months
NCT02149225	Personalized peptide	APVAC1, APVAC2	SOC	1	nGBM	Tolerable safety. mPFS: 14.2 months, mOS: 29 months
NCT02287428	Personalized peptide	PSLP	RT, pembrolizumab	1	nGBM	Ongoing.
UMIN1426	Personalized Peptide	AFTV	SOC	2b	nGBM	mOS: 25.6 months for AFTV vs. 31.5 months for placebo; mpFS 13.3 months in both groups. Total tumor removal: 3-year OS: 80% for AFTV vs. 54% placebo; 3-year PFS 81% for AFTV vs. 46% placebo.
jRCT2031200153	Personalized Peptide	AFTV	SOC including GTR	3	nGBM	Ongoing.
NCT02718443	Nucleic acid	VXM01	+/− surgery; +/− nivolumab	1	rGBM	Tolerable safety. 12-mOS: 7/12 patients (58.3%)
NCT03750071	Nucleic acid	VXM01	Avelumab	1/2	rGBM	Tolerable safety. ORR 12%
NCT00905060	HSP	HSPPC-96	SOC	2	nGBM	Tolerable safety. mOS: 23.8 months
NCT00293423	HSP	HSPPC-96	GTR	2	rGBM	Tolerable safety. mOS: 42.6 weeks

** Trial identification unavailable on ClinicalTrials.gov and CTIS; DC = dendritic cell; SOC = standard of care; TMZ = temozolomide; nGBM = newly diagnosed GBM; rGBM = recurrent GBM; GTR = gross total resection; mPFS = median progression-free survival; mOS = median overall survival; ORR = objective response rate; CMV = cytomegalovirus; ALT = autologous lymphocyte transfer; ACT= adoptive cellular therapies; rMB = recurrent medulloblastoma; HSP = heat shock protein.

**Table 4:** Clinical trials evaluating CAR T cells for the treatment of high-grade gliomas.

Trial ID	CAR-T Target	Phase	Tumor Type(s)	Outcomes/Observational Data
NCT02208362	IL-13Rα2	1	rHGG	Tolerable safety. rGBM: Dual delivery (intra-tumoral/intraventricular) route: mOS 10.2 months, vs. 7.7 months for all patients with recurrent GBM
NCT02209376	EGFRvIII	1	rGBM	Tolerable safety. mOS: 251 days.
NCT05660369	EGFRvIII, wild-type EGFR	1	nGBM, rGBM	Tolerable safety. Transient radiographic improvement in 2 patients (*n* = 3).
NCT05168423	EGFR, IL-13Rα2	1	rGBM	Early-onset neurotoxicity was observed but manageable. 1 patient in DL2 developed DLT. None met criteria for ORR.
NCT04196413	GD2	1	H3K27 mutant tumors (DIPG, sDPG)	Tolerable safety. Major volumetric reduction (>50%, *n* = 4/9), CR for >30 months (*n* = 1), neurological benefit (9/9).

rHGG = Recurrent high-grade glioma; rGBM = recurrent GBM; nGBM = newly diagnosed GBM; DIPG = diffuse intrinsic pontine glioma; DL2 = dose level 2; DLT = dose-limiting toxicity; mOS = median overall survival; ORR = objective response rate; CR = complete response.

**Table 5:** Clinical Trials Evaluating Cytokine Therapies for the Treatment of High-Grade Gliomas.

Trial ID	Cytokine	Additional Treatments	Phase	Tumor Type(s)	Outcomes
NCT01765088	IFN-α	SOC	3	nHGG	mOS: 26.7 months for adjuvant TMZ + IFN-α, vs. 18.8 months for SOC with adjuvant TMZ alone
**	IFN-α	RT + carmustine	3	nHGG	No significant difference in survival or response rates.
NCT04443010	TNF (L19TNF)	SOC	1	nGBM	Ongoing.
NCT04573192	TNF (L19TNF)	Lomustine	1/2	rGBM	Ongoing.
NCT03687957	IL-7 (NT-I7)	SOC	1	nHGG	Tolerable safety.
NCT05465954	IL-7 (NT-I7)	Surgery + pembrolizumab	2	rGBM	Ongoing.

** = Trial identifier not available on clinicaltrials.gov or CTIS; SOC = standard of care; nHGG = newly diagnosed high-grade glioma; nGBM = newly diagnosed glioblastoma; rGBM = recurrent glioblastoma; mOS = median overall survival; TMZ = temozolomide; RT = radiation therapy.
